# Jean Pierre Mégnin (1828–1905): A Nineteenth-Century Pioneer in the Development of Veterinary and Forensic Sciences

**DOI:** 10.7759/cureus.85055

**Published:** 2025-05-29

**Authors:** Ioannis Nikolakakis, Angeliki Geronymou, Ifigeneia Avgousti, Marianna Karamanou, Konstantinos Moraitis

**Affiliations:** 1 Emergency Department, Tzaneio Prefecture General Hospital of Piraeus, Athens, GRC; 2 Department of History of Medicine and Medical Ethics, National and Kapodistrian University of Athens School of Medicine, Athens, GRC; 3 Department of Forensic Medicine and Toxicology, National and Kapodistrian University of Athens School of Medicine, Athens, GRC

**Keywords:** clinical forensic medicine, forensic assessment, forensic evidence, forensic science, historical vignette

## Abstract

Jean Pierre Mégnin (1828-1905) was a pathbreaking veterinary physician and entomologist who made fundamental contributions to forensic science. Mégnin, born in 1828, began his career in the military before becoming a veterinarian. He laid the foundation of forensic entomology when he became interested in studying the role of insects in the decomposition of animal and human remains. His scientific contributions to funerary practices included estimating burial time based on insect and mite colonization patterns in cadaverous remains. Furthermore, Mégnin made a significant contribution to veterinary science in the fields of acarology, parasitology, and differential diagnosis of dog breeds. His 1894 work, *La Faune des Cadavres *(*The Fauna of Corpses*), detailed the ecological succession of insects on corpses and profoundly changed the forensic practitioner’s approach to death. Today, Mégnin’s contribution remains highly relevant, especially in the fields of forensic science, forensic entomology, and taphonomy.

## Introduction and background

**Figure 1:**
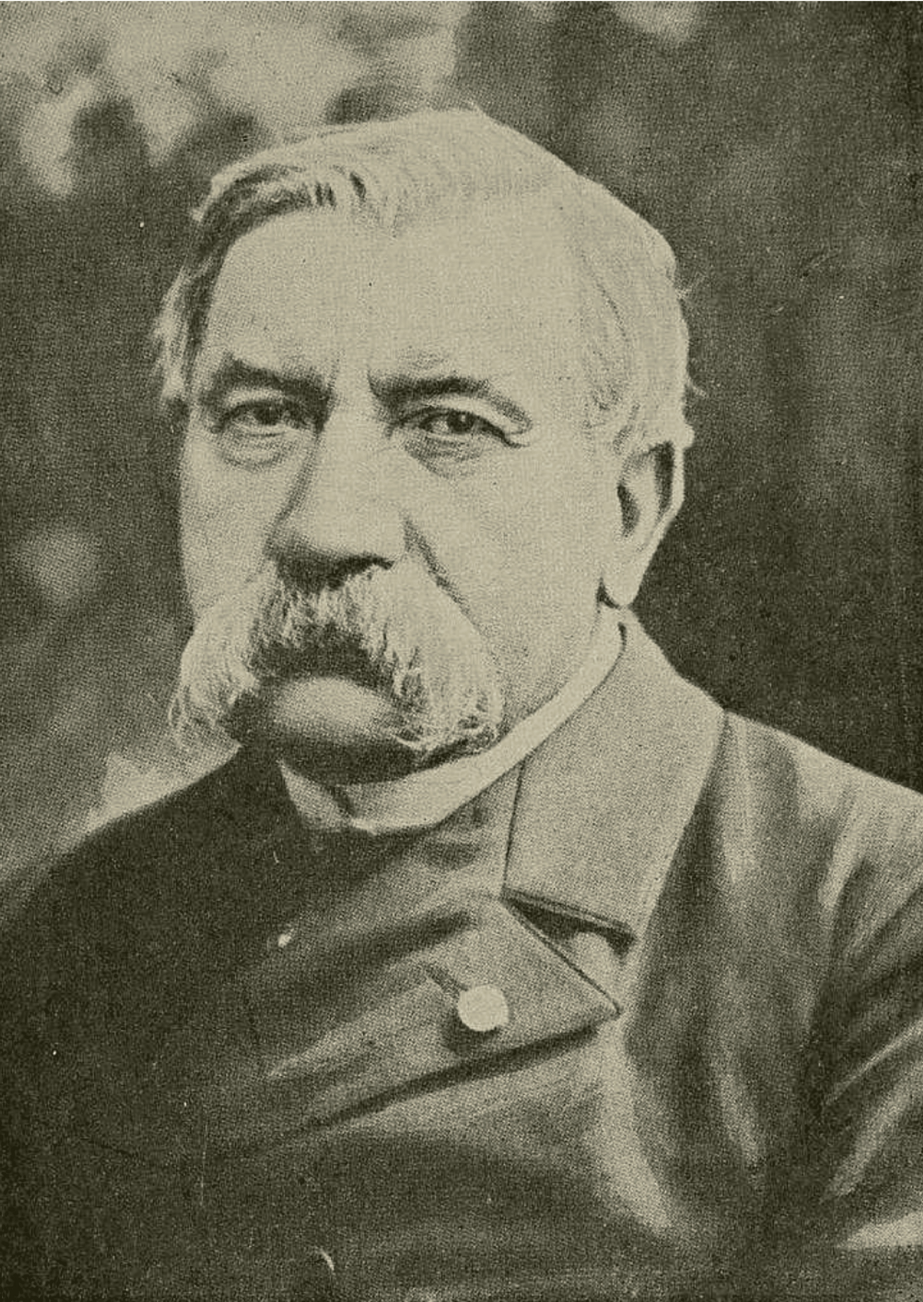
Jean Pierre Mégnin (1885) Image in the public domain via Wikimedia Commons.

Jean Pierre Mégnin (1828-1905) (Figure 1) was a pioneering French veterinarian and entomologist, recognized as one of the first scientists who had systematically studied the role of arthropods in forensic investigations [1]. After studying veterinary medicine in Paris, he served in various distinguished units of the French military and applied his research on the battlefield [2]. Ηe then focused on the application of entomology and acarology to legal medicine. His groundbreaking work *La faune des cadavres* (1894) introduced the concept of eight successive waves of insect colonization on decomposing corpses, which became foundational in the estimation of post-mortem intervals. Mégnin’s studies on body mites and other related issues were considered foundational for the development of forensic acarology. As a result, his theories significantly influenced the methods applied at crime scenes and promoted the early foundations of forensic taphonomy. Through careful observations and detailed case studies, Mégnin substantiated his views on forensic entomology and its effectiveness in legal medicine, earning a reputation as a pioneer of the discipline [1].

## Review

Early life and career

Jean Pierre Mégnin was born on January 16, 1828, in Hérimoncourt, a commune located in the Bourgogne-Franche-Comté region. He became interested in the natural sciences and, in 1849, enrolled at the distinguished École d'Alfort, the Veterinary School of Medicine in Paris. Four years later, in 1853, he was accepted as a veterinarian and in 1855 entered the Imperial Cavalry School in Saumur, joining the 2nd Artillery Regiment. He later served in distinguished units, including the Imperial Guard's Mounted Artillery in 1864, the 15th Dragoons Regiment in 1871, and the 12th Artillery Regiment, where he remained until his retirement [2].

His time on the battlefield inspired him to study the decomposition of human bodies by insects, which influenced his work in forensic entomology. He focused on estimating the time of death based on the species that colonize bodies and their activities. Mégnin was also deeply involved in scientific research and writing. His interest in the scientific domain was sparked during his work as a veterinarian, where he observed the behavioral actions of dogs, horses, and insects, which he illustrated by developing diagrams of their life cycle. He was motivated by a desire to understand and help alleviate everyday challenges through scientific methods. This interest led to further studies and notable contributions to forensic entomology [3,4].

In addition to his scientific activities, Mégnin was also involved in publishing and teaching. He founded and edited *L'Éleveur*, a scientific journal on animal husbandry, which allowed him to share knowledge and be recognized in veterinary science and entomology [5].

Publications and legacy

One of Mégnin's earliest notable works, entitled *Maladies parasitaires chez l'homme et les animaux domestiques* (Parasitic Diseases in Humans and Domestic Animals) (1880), is an exhaustive review of the knowledge of parasitic diseases affecting both humans and domestic animals. The extensive erudition within the field of parasitology was evident in this publication. Mégnin’s work in parasitology contributed to understanding how certain parasites affect both human and animal health [4].

Mégnin’s meticulous work on acarology - particularly on the significant feather mites of domestic chickens belonging to the family of Analgidae - led to the naming of a genus in his honor. The genus* Megninia* includes *Megninia ginglymura*, described by Mégnin in 1877. This feather mite is found in Europe, Africa, Asia, and the Americas, having caused severe economic losses in commercial poultry farms at that time, reducing egg production and leading to poultry loss. Another essential species affecting domestic poultry is *Megninia cubitalis* [6,7,8]. In addition, the spinose ear tick species *Otobius megnini* was named in 1883 by physician Alfred Dugès (1826-1910) in honor of his former teacher, Jean Pierre Mégnin. Originally described as *Argas megnini*, this species further illustrates how Mégnin’s pioneering contributions to parasitology and veterinary science have endured through time [9].

Jean Pierre Mégnin was the first to use the name “beauceron”, replacing the previous names of long-haired and short-haired shepherds' canines: Berger de la Brie and Berger de la Beauce, respectively [10]. In 1897, he also introduced the first classification system for dog breeds, dividing them into four main types based on their morphology: mollossoid (heavy-boned), lupoid (wolf-type), braccoid (hound-type), and graioid (greyhounds) [11].

Another significant contribution is his 1888 publication* Faune des tombeaux* (Fauna of the Tombs) in the journal *La Nature*, which details the fauna associated with graves. This work is vital because it was a forerunner of his later forensic publication, *La faune des cadavres*, and laid the foundation for the application of entomology in legal settings. In his work, Mégnin, who was working on Acari, began exploring the idea that specific types of insects are attracted to decomposing bodies, a concept he further developed in his subsequent books. This idea became a cornerstone of forensic entomology [12,13].

Jean Pierre Mégnin was pivotal in the early development of forensic acarology. In 1894,* La faune des cadavres* - his most renowned book - was published, in which he described the colonization patterns of insects on decaying bodies [14]. In this work, he revised his earlier theory of four insect waves infesting bodies exposed outdoors, expanding it to include eight successional waves. These eight specific successional waves of insects depict the phases in which the insects arrive at the body, inhabit it for a certain period, and then depart. The initial wave comprised flies and mites, while the sixth wave consisted solely of mites [15]. These phases of body colonization, during which insects feed for a certain period of time as the body progressively decomposes, biochemically changing the corpse. These alterations render the body unattractive to certain insect groups, prompting colonization by a different set of insects [3,16].

As far as the buried bodies are concerned, he mentioned two waves. The book presents both larval and adult forms from numerous families. Its illustrations primarily depict wing venation, posterior spiracles, and the anatomical features used in species identification. He also included 19 case reports, some of which were his own from 1879 to 1888. His original statements, as cited in court, are presented alongside the most significant inquiries he received as an expert witness [17]. He formulated the first official definition and a testable model of ecological succession, highlighting the predictable sequences of changes involving carrion insects and the distribution of resources among human bodies. These discoveries played a crucial role in forensic science. His research laid the foundation for two major fields: carrion ecology and forensic entomology [18]. Mégnin was among the first to integrate forensic entomology into the broader discipline of legal medicine, contributing to a systematic approach to crime scene investigation involving decomposition [14].

Τhere are numerous citations to his work in historical and contemporary forensic literature, underlining that it is still remarkably pertinent today. Jean Pierre Mégnin was acknowledged during his lifetime and received many distinctions, including the Legion of Honor [2].

Contribution to forensic sciences

Mégnin’s significant contribution to forensic science was his research on the sequence of insect activity on bodies, revolutionizing the estimation of the time since death. A milestone in Mégnin’s career was his forensic investigation into the case of the mummified newborn baby girl in Paris, France, in 1878. The estimation of the time of death was based on the insects and mites that had colonized the body, along with the forensic pathologist’s autopsy findings. This was the first case where the mites contributed to estimating the postmortem interval (PMI) [19]. Moreover, it was the second case in the history of forensic entomology in France. The first was reported in 1855 by Dr. Bergeret, who used blow fly pupae and larval moths for his estimations [17]. The insects recovered from the body were mainly mites and some caterpillars. Mégnin identified these mites as *Tyroglyphus longior* and proposed that they had reached the body by phoresis, hitching a ride on carrier insects. He calculated that the colonization had begun approximately five months before the autopsy [19].

Although Mégnin was a highly reputable acarologist, there is still debate about his findings in this case. Firstly, *Tyrophagus* mites are non-phoretic, meaning that their arrival may have occurred almost immediately after the newborn’s death, as this type of mite typically lives in the soil and grassland. If they were phoretic forms, colonization would have been delayed due to the additional time required for their carriers to arrive at the body. In addition, temperature was not factored into Mégnin’s calculation of the mite population growth rate. Considering the weather conditions of 1877 and 1878, as well as the biology of these mites, it is believed that the *Tyrophagus* may have reached the body almost immediately after death and that the colony could have multiplied over the span of eight months. This conclusion does not align with Mégnin’s estimate of five months prior to the body’s discovery, and the case is still considered unsolved [19].

One of his most remarkable contributions to forensic science was initiating work in what is now recognized as forensic taphonomy, the study of all processes affecting organic remains after death. His research, particularly on the role of insects during decomposition, significantly improved the accuracy of PMI estimation. He emphasized the importance of integrating multiple scientific disciplines, offering new insights into the postmortem alterations of human remains. These subject areas continue to shape and advance forensic entomology, especially in complex cases involving a multidisciplinary approach [16]. Despite contemporary forensic entomology expanding on his “eight waves” methodology, Mégnin’s 1894 monograph *La Faune des Cadavres* still serves as the classic reference for all subsequent advancements.

## Conclusions

Jean Pierre Mégnin’s innovative research in forensic entomology and acarology positioned him as one of the pioneers at the intersection of forensic and entomological sciences. His early research on the effects of insects in human decomposition laid the foundation for time since death estimation procedures, which have become standard in contemporary forensic investigations. Mégnin’s interdisciplinary approach, along with his meticulous attention to the systematic description of the insects’ role in forensics, contributed to the development of forensic taphonomy as a distinct field. His research also advanced veterinary sciences and had far-reaching consequences for legal medicine and forensic practice. The legacy of his work continues to influence the development of forensic sciences and remains relevant in contemporary forensic cases.
